# Asperlones A and B, Dinaphthalenone Derivatives from a Mangrove Endophytic Fungus *Aspergillus* sp. 16-5C

**DOI:** 10.3390/md13010366

**Published:** 2015-01-13

**Authors:** Ze’en Xiao, Shao’e Lin, Chunbing Tan, Yongjun Lu, Lei He, Xishan Huang, Zhigang She

**Affiliations:** 1 School of Chemistry and Chemical Engineering, Sun Yat-sen University, Guangzhou 510275, China; E-Mails: xiaozeen@mail2.sysu.edu.cn (Z.X.); linshaoe@mail2.sysu.edu.cn (S.L.); 15872431502@163.com (C.T.); 2 School of Life Sciences and Biomedical Center, Sun Yat-sen University, Guangzhou 510275, China; E-Mails: luyj@mail.sysu.edu.cn (Y.L.); helei8688@126.com (L.H.); 3 Guangdong Province Key Laboratory of Functional Molecules in Oceanic Microorganism, Bureau of Education, Sun Yat-sen University, 74 Zhongshan Road II, Guangzhou 510080, China

**Keywords:** marine fungi, *Aspergillus* sp., asperlones, azaphilones, MptpB inhibitor

## Abstract

Racemic dinaphthalenone derivatives, (±)-asperlone A (**1**) and (±)-asperlone B (**2**), and two new azaphilones, 6″-hydroxy-(*R*)-mitorubrinic acid (**3**) and purpurquinone D (**4**), along with four known compounds, (−)-mitorubrinic acid (**5**), (−)-mitorubrin (**6**), purpurquinone A (**7**) and orsellinic acid (**8**), were isolated from the cultures of *Aspergillus* sp. 16-5C. The structures were elucidated using comprehensive spectroscopic methods, including 1D and 2D NMR spectra and the structures of **1** further confirmed by single-crystal X-ray diffraction analysis, while the absolute configuration of **3** and **4** were determined by comparing their optical rotation and CD with those of the literature, respectively. Compounds **1**, **2** and **6** exhibited potent inhibitory effects against *Mycobacterium tuberculosis* protein tyrosine phosphatase B (MptpB) with IC_50_ values of 4.24 ± 0.41, 4.32 ± 0.60 and 3.99 ± 0.34 μM, respectively.

## 1. Introduction

Tuberculosis (TB) ranks as the second leading cause of death from an infectious disease worldwide; an estimated 9.0 million people developed TB and 1.5 million died from the disease in 2013, according to the WHO [1]. In recent years, extensively drug-resistant TB (DR-TB), multidrug-resistant TB (MDR-TB) and HIV-associated TB have made clinical treatment even more difficult and complex. New chemotherapeutic approaches and unusual anti-infective agents are in urgent need, especially those applying to new targets and based on different mechanisms. *Mycobacterium tuberculosis* protein tyrosine phosphatase B (MptpB) is secreted by the microbe and manipulates host signal transduction pathways, which has proven to be an essential virulence factor when *M. tuberculosis* hosts macrophages [2,3,4,5,6]. Increased research reveals that it exhibits unique and multiple activities against immune responses [7,8,9,10,11,12,13]. Therefore, finding new inhibitors of MptpB could be a promising strategy against *M. tuberculosis* infection and conducive to the treatment of TB.

**Scheme 1:**
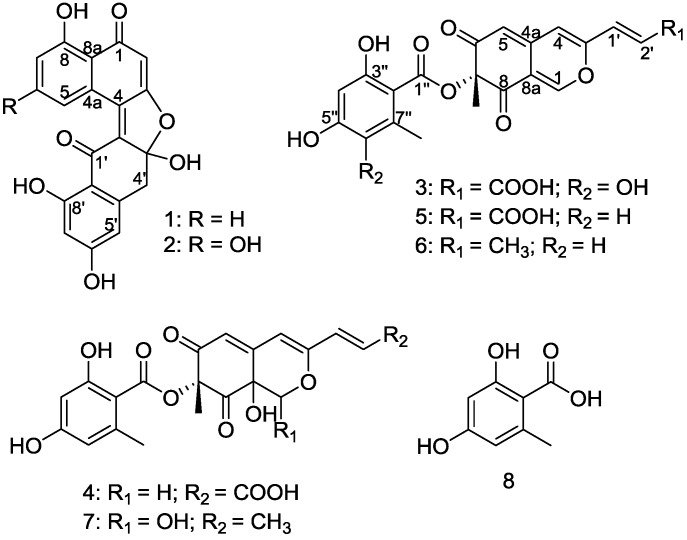
Chemical structures of compounds (**1**)–(**8**).

As part of our ongoing investigation on unusual biological activity compounds from mangrove endophytic fungi collected from the South China Sea [14,15,16,17,18,19,20], a mangrove endophytic fungus, named *Aspergillus* sp. 16-5C, attracted our attention. During the course of our investigation on the chemical constituents from the fungus, four new compounds, (±)-asperlones A (**1**) and B (**2**), 6″-hydroxy-(*R*)-mitorubrinic acid (**3**) and purpurquinone D (**4**), together with four known compounds, (−)-mitorubrinic acid (**5**), (−)-mitorubrin (**6**), purpurquinone A (**7**) and orsellinic acid (**8**) (Scheme 1), were isolated. In this report, we describe the isolation, structural elucidation, biosynthetic pathways and biological activity of the metabolites.

## 2. Results and Discussion

**Figure 1:**
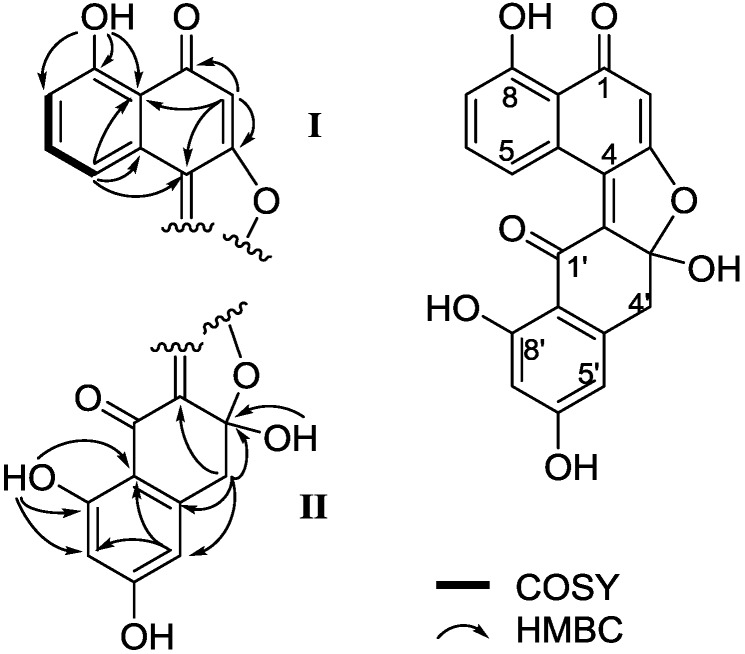
COSY and key HMBC correlations of (±)-asperlone A.

**Figure 2:**
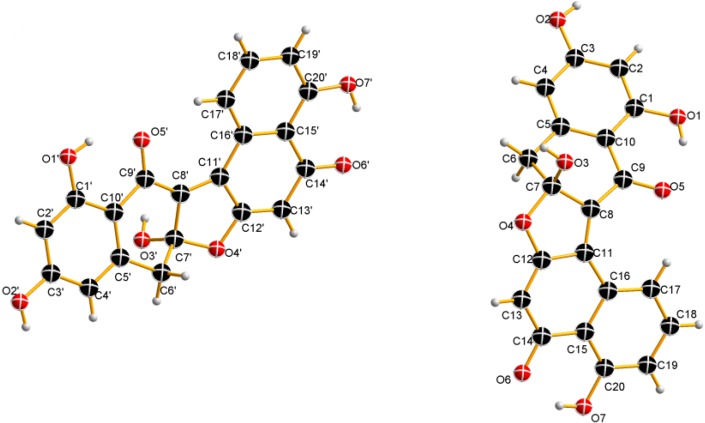
Perspective ORTEP illustrations of (±)-asperlone A.

(±)-Asperlone A (**1**) was obtained as a red crystal (MeOH). The molecular formula C_20_H_12_O_7_ results from the HR-ESI mass spectrum (*m*/*z* = 363.0512, [M − H]^−^) and indicates 15 degrees of unsaturation. The presence of hydroxyl and carbonyl groups is shown by IR absorbtion bands at ν_max_ 3394 and 1647 cm^−1^. The ^1^H NMR spectrum exhibits 12 proton signals in DMSO-*d*_6_, which revealed the presence of six aromatic/quinoid protons (δ_H_ 6.17, 6.25, 6.40, 7.20, 7.68 and 8.80), a methylene group (δ_H_ 3.41/3.58) and four exchangeable protons (δ_H_ 8.23, 11.30, 12.84, 13.58). The ^13^C NMR spectrum exhibits 20 carbon signals (Table 1). Besides the proton attached carbon atoms, the signals of 13 quaternary carbon atoms are observable, including two carbonyl groups (δ_C_ 186.7 and 191.8). Proton and carbon signal assignments result from the HSQC. Interpretation of the ^1^H–^1^H COSY indicated the presence of one substructure (bolded lines in Figure 1). In the HMBC spectrum, correlations from the quinoid proton at δ_H_ 6.17 (H-2) to C-1 (δ_C_ 191.8), C-3 (δ_C_ 171.6), C-4 (δ_C_ 129.8), C-8a (δ_C_ 113.8) and from aromatic proton at δ_H_ 8.80 (H-5) to C-4, C-4a (δ_C_ 128.4) and C-8a established the structure of fragment **I**. HMBC correlation from the signal at δ_H_ 8.23 (OH-3′) to the quaternary carbon signal (δ_C_ 113.5) suggested that C-3′ is a hemiacetal carbon. Furthermore, HMBC correlations from the methylene signals at δ_H_ 3.41/3.58 (H2-4′) to C-2′ (δ_C_ 142.7), C-3′, C-4a′ (δ_C_ 144.0), C-5′ (δ_C_ 110.4), from the exchangeable proton at δ_H_ 12.84 (OH-8′) to C-7′ (δ_C_ 101.6), C-8′ (δ_C_ 167.3), C-8a′ (δ_C_ 112.0), and from the aromatic proton at δ_H_ 6.40 (H-5′) to C-7′ and C-8a′ established the structure of fragment **II**. Taken together, the planar structure of **1** was defined (Figure 1). No CD (circular dichroism) spectrum could be obtained from **1**, indicating a racemic mixture of the possible enantiomers, which results from the center of chirality (C-3′) of the hemiketal. Ultimately, the structure of **1** was subsequently confirmed by single-crystal X-ray diffraction experiments using Cu Kα radiation (Figure 2), named (±)-asperlone A. (±)-Asperlone A were attempted to separate by chiral HPLC and using four types of chiral columns, but all were unsuccessful.

**Table 1:** ^13^C NMR (100 MHz) and ^1^H NMR (400 MHz) data of **1** and **2** (DMSO-*d*_6_).

Position	1		2	
	δ_C_, mult.	δ_H_ (*J* in Hz)	δ_C_, mult.	δ_H_ (*J* in Hz)
1	191.8, qC		190.5, qC	
2	102.1, CH	6.17 s	101.6, CH	6.03 s
3	171.6, qC		170.6, qC	
4	129.8, qC		130.2, qC	
4a	128.4, qC		129.8, qC	
5	121.8, CH	8.80 d (7.8)	111.2, CH	8.34 d (2.2)
6	135.3, CH	7.68 m	163.4, qC	
7	122.5, CH	7.20 d (8.3)	106.7, CH	6.47 d (2.2)
8	162.1, qC		164.4, qC	
8a	113.8, qC		107.2, qC	
1′	186.7, qC		186.5, qC	
2′	142.7, qC		142.2, qC	
3′	113.5, qC		113.2, qC	
4′	41.0, CH_2_	3.41 d (15.7); 3.58 d (15.7)	41.1, CH_2_	3.39 d (15.8); 3.55 d (15.8)
4a′	144.0, qC		144.0, qC	
5′	110.4, CH	6.40 d (1.6)	110.4, CH	6.40 d (1.6)
6′	167.1, qC		167.1, qC	
7′	101.6, CH	6.25 d (1.6)	101.6, CH	6.25 d (1.6)
8′	167.3, qC		167.0, qC	
8a′	112.0, qC		112.0, qC	
6-OH				10.83 brs
8-OH		13.58 s		13.76 s
3′-OH		8.23 brs		8.14 brs
6′-OH		11.30 brs		11.17 brs
8′-OH		12.84 s		12.93 s

(±)-Asperlone B (**2**), isolated as a red amorphous powder, exhibits one oxygen more than **1**, resulting in the molecular formula C_20_H_12_O_8_ (HR-ESIMS *m*/*z* = 379.0461, [M − H]^−^). The close resemblance between the NMR spectra of **1** and **2** indicated that **2** was another dinaphthalenone derivative and the major difference was the ^13^C NMR chemical shift of C-6 increased from 135.3 to 163.4, while the ^1^H NMR chemical shifts of H-5/7 (δ_H_ 8.80 (d, 7.8)/ 7.20 (d, 8.3)) reduced to 8.34 (d, 2.2) and 6.47 (d, 2.2), respectively. The aromatic proton in **1** (δ_H_ 7.68, H-6) was replaced by a hydroxyl in **2** (δ_H_ 10.83), which suggested that **2** is a 6-oxygenated derivative of **1**. The HMBC correlations from H-5 (δ_H_ 8.34) and H-7 (δ_H_ 6.47) to this aromatic carbon (δ_C_ 163.4) further confirmed that **2** was 6-hydroxyasperlone A. The absence of any CD spectrum indicating that **2** is also a racemic mixture. Unfortunately, the single crystal of compound **2** was unable to be obtained and the structure was named (±)-asperlone B. Resolution of the separation of **2** was also unsuccessful.

In most cases, natural products are produced in optically pure form, with only one enantiomer biosynthesized. Enantiomerically opposite products are also metabolized, but at a rare occurrence of less than 1% relative to the overall abundance of natural products, which often result from the action of stereochemically distinct enzymes that can give single and opposite enantiomeric products from achiral substrates [21,22].

**Figure 3:**
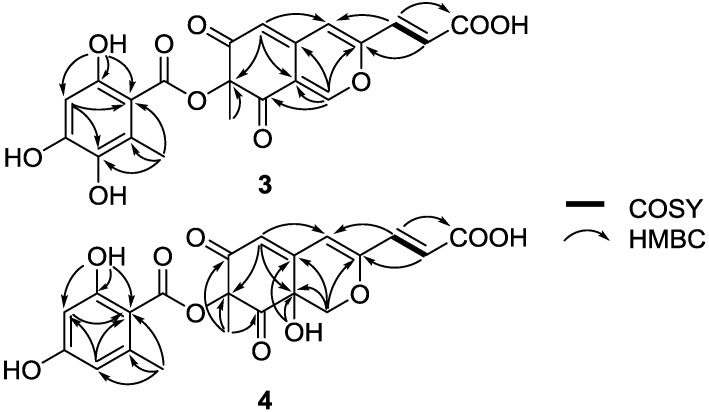
COSY and key HMBC correlations of **3** and **4**.

6″-Hydroxy-(*R*)-mitorubrinic acid (**3**) isolated as a yellow power was assigned the molecular formula C_21_H_16_O_10_ on the basis of HR-ESIMS (*m*/*z* = 427.0669, [M − H]^−^), consistent with 14 degrees of unsaturation. The IR spectrum showed the presence of a hydroxy (3425 cm^−1^) and a conjugated carbonyl (1722 and 1624 cm^−1^). The ^1^H NMR spectrum (Table 2) showed six olefinic protons (δ_H_ 5.73, 6.24, 6.43, 7.14, 7.28 and 8.32), four hydroxy signals (δ_H_ 8.00, 9.68, 10.28, and 12.86) and two methyl signals (δ_H_ 1.56 and 2.36). In addition, the ^13^C NMR spectrum exhibits two carbonyl groups (δ_C_ 192.2 and 192.6), suggesting the presence of an azaphilone core and an orsellinic acid [23] moiety for **3**, analogous to (−)-mitorubrinic acid (**5**) [24]. The differences in NMR data between **3** and **5** could be explained by the replacement of an aromatic proton in **5** with a phenolic hydroxyl group (δ_H_ 8.00) in **3**, indicating that **3** is the hydroxy derivative of **5**. The key HMBC correlations from H-4″ (δ_H_ 6.24) and CH_3_-7″ (δ_H_ 2.36) to C-6″ (δ_C_ 137.3) suggested that the hydroxylation occurs at C-6″ (Figure 3). The optical rotation of ${\left[\alpha \right]}_{D}^{20}$ = −267.8 clearly revealed the *7R*-configuration, as same as **5**. Thus, the structure of **3** was elucidated as 6″-hydroxy-(*R*)-mitorubrinic acid. 

**Table 2:** ^13^C NMR (100 MHz) and ^1^H NMR (400 MHz) data of **3** and **4** (DMSO-*d*_6_).

Position	3		4	
	δ_C_, mult.	δ_H_ (*J* in Hz)	δ_C_	δ_H_ (*J* in Hz)
1	155.5, CH	8.32 s	69.8, CH_2_	3.96 d (12.4); 4.68 d (12.4)
3	153.3, qC		156.7, qC	
4	116.2, CH	7.14 s	109.1, CH	6.46 s
4a	142.4, qC		148.1, qC	
5	109.2, CH	5.73 s	120.2, CH	6.21 s
6	192.2, qC		192.2, qC	
7	85.5, qC		84.1, qC	
7-CH_3_	22.3, CH_3_	1.56 s	23.9, CH_2_	1.82 s
8	192.6, qC		200.0, qC	
8a	114.7, qC		66.2, qC	
1′	134.0, CH	7.28 d (15.7)	136.7, CH	7.13 d (15.6)
2′	125.0, CH	6.43 d (15.7)	124.2, CH	6.34 d (15.6)
3′	166.6, qC		167.0, qC	
1″	168.7, qC		168.2, qC	
2″	105.0, qC		105.1, qC	
3″	155.2, qC		163.1, qC	
4″	101.0, CH	6.24 s	101.0, CH	6.16 d (2.0)
5″	152.2, qC		162.8, qC	
6″	137.3, qC		111.6, CH	6.24 d (2.0)
7″	126.2, qC		142.8, qC	
7″-CH_3_	14.6, CH_2_	2.36 s	23.1, CH_2_	2.44 s
8a-OH				7.26 s
3′-OH		12.86 brs		12.82 brs
3″-OH		9.68 s		10.26 s
5″-OH		10.28 brs		10.32 brs
6″-OH		8.00 brs		

**Figure 4:**
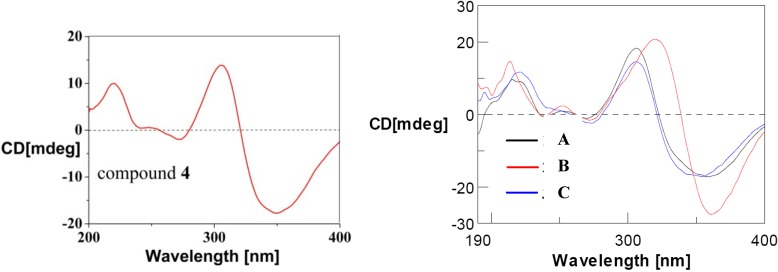
CD curves of compound **4** and purpurquinones A–C.

Purpurquinone D (**4**) was obtained as a yellowish powder and was assigned the molecular formula C_21_H_18_O_10_ from HR-ESIMS (*m*/*z* = 429.0824, [M − H]^−^), with one oxygen atom and two hydrogen atoms more than that of **5** [24]. Analysis of the NMR data for **4** (Table 2) revealed the presence of similar structural features to those found in **5**, except that an olefinic double bond was replaced by a methylene (δ_H_/δ_C_ 3.96, 4.68/69.8) and an oxygenated quaternary carbon (δ_C_ 66.2), indicating that **4** is the hydroxylated derivative of **5**. In addition, the key HMBC correlations from OH-8a (δ_H_ 7.26) to C-4a/8a/1 (δ_C_ 148.1/66.2/69.8), and from H2-1 (δ_H_ 3.96, 4.68) to C-4a/8a/3 (δ_C_ 148.1/66.2/156.7) revealed that the hydration occurs at C_1_–C_8a_ double bond (Figure 3). The NOESY correlations among OH-8a and H3-7 (δ_H_ 1.82) suggested a *cis*-configuration between OH-8a and H3-7. Further more, the similar CD Cotton effects (Figure 4) at 350 nm (Δε −23.1), 305 nm (Δε +18.1), and 220 nm (Δε +13.0) indicated that **4** has the same (*7R*, *8aS*)-configuration as purpurquinone C [25]. Thus, the structure of **4** was identified and named purpurquinone D.

The remaining four known compounds were identified as (−)-mitorubrinic acid (**5**) [24], (−)-mitorubrin (**6**) [24], purpurquinone A (**7**) [25] and orsellinic acid (**8**) [23], respectively, by comparison of their NMR and MS data with those reported.

**Scheme 2:**
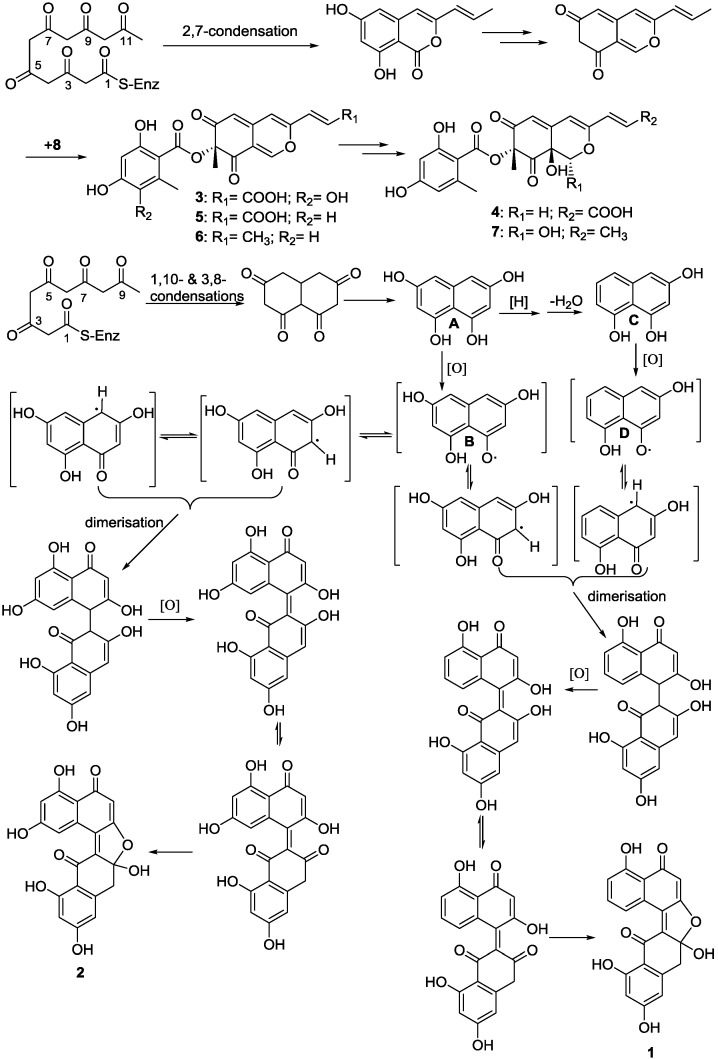
Proposed biosynthetic pathways for compounds (**1**)–(**7**).

In light of our hypothesis that all these compounds are the product of a fungal polyketide synthase [26]. The unusual dinaphthalenone derivatives, named (±)-asperlones A (**1**) and B (**2**), are presumed to be started from five acetate units through a series of polyketide biosynthesis, reduction, dehydration, oxidation and dimerization [27,28,29], as shown in Scheme 2. The initial condensations of tetraoxodecanoic acid and further reduction leading to compound **A**, followed by oxidation to generate compound **B** and subsequent dimerization, oxidation and rearrangement to generate (±)-asperlone B (**2**). On the other way, the reduction and dehydration of **A** leading to **C** and further oxidation to generate **D**. Subsequent dimerization of the rearrangement structure of **B** and **D** afford the dinaphthalenone skeleton and then further oxidize and rearrange to generate (±)-asperlone A (**1**).

The activity of compounds **1**–**3** and **5**–**6** against MptpB was evaluated with sodium orthovanadate as the positive control (Table 3). The results showed that compounds (±)-**1**, (±)-**2** and **6** were strong inhibitors of of MptpB with IC_50_ value of 4.24 ± 0.41, 4.32 ± 0.60 and 3.99 ± 0.34 μM, respectively, which revealed that isolated compounds **1**, **2** and **6** could be potential antituberculosis drugs.

**Table 3:** *Mycobacterium tuberculosis* protein tyrosine phosphatase B (MptpB) assay for **1**–**3** and **5**–**6**.

Compound	(±)-1	(±)-2	3	5	6	Positive Control
IC_50_ (μM)	4.24 ± 0.41	4.32 ± 0.60	>50	>50	3.99 ± 0.34	0.05

## 3. Experimental Section

### 3.1. General

Melting points were measured on an X-4 micromeltin-point apparatus (Cany Precision Instruments Co., Ltd., Shanghai, China) and are uncorrected. Optical rotations were determined with a RUDOLPH Autopol III polarimeter (Rudolph Research Analytical, Hackettstown, NJ, USA) at 20 °C. UV data were measured on a PERSEE TU-1900 spectrophotometer (Purkinje General Instrument Co., Ltd., Beijing, China). IR spectra were measured on a Nicolet Nexus 670 spectrophotometer (Thermo Fisher Scientific, Inc., Hudson, NH, USA), in KBr discs. CD data were measured on a Chirascan™ CD spectrometer (Applied Photophysics, London, UK). ^1^H and ^13^C NMR data were recorded on a Bruker AVANCE 400 spectrometer (Bruker BioSpin Corporation, Billerica, MA, USA) in DMSO-*d*_6_. ESIMS spectra were obtained from a Micro mass Q-TOF spectrometer (Waters Corporation, Milford, MA, USA). HR-ESIMS spectra were measured on a Thermo Scientific LTQ Orbitrap Elite high-resolution mass spectrometer (Thermo Fisher Scientific, Inc., Hudson, NH, USA). Silica gel (Qing Dao Hai Yang Chemical Group Co., Qingdao, China; 200–300 mesh), octadecylsilyl silica gel (Unicorn, Palarivattom, Kerala, India; 45 μm) and Sephadex LH-20 (GE Healthcare, Buckinghamshire, UK) were used for column chromatography. Precoated silica gel plates (Qing Dao Huang Hai Chemical Group Co., Qingdao, China; G60, F-254) were used for thin layer chromatography.

### 3.2. Fungal Material

The fungus used in this study was isolated from a mangrove endophytic fungus from the leaves of *S. apetala*, which were collected in Hainan Island, China. The fungus was identified as *Aspergillus* sp. according to a molecular biological protocol by DNA amplification and sequencing of the ITS region (deposited in GenBank, accession No. JX993829). A voucher strain was deposited in School of Chemistry and Chemical Engineering, Sun Yat-sen University, Guangzhou, China, with the access code, 16-5C.

### 3.3. Extraction and Isolation

The fungal strain *Aspergillus* sp. 16-5C was cultivated in potato glucose liquid medium (15 g glucose and 3 g sea salt in 1 L potato infusion) in 1 L Erlenmeyer flasks, each containing 300 mL of culture broth, at 28 °C without shaking for 30 days. The culture (50 L) was filtered to separate the culture broth from the mycelia, and then the mycelia were extracted three times with EtOAc. The organic solvent was filtered and concentrated under reduced pressure to yield 4.7 g of organic extract, which was subjected to silica gel column chromatography (CC) (petroleum ether-EtOAc from 90:10 to 0:100 (v/v), gradient) to generate six fractions (Fr. 1–6). Fr.2 was further purified by silica gel CC using 30% EtOAc-light petroleum, then subjected to Sephadex LH-20 CC eluting with CHCl_3_/MeOH (1:1) to obtain compounds **1** (1.0 mg) and **2** (1.5 mg). Meanwhile, Fr. 4 was further purified silica gel CC using 40% EtOAc-light petroleum to afford seven subfractions (Frs. 4.1–4.7). Fr.4.3 was applied to Sephadex LH-20 CC, eluted with CHCl_3_/MeOH (1:1), to obtain two subfractions (Frs. 4.3.1–4.3.2). Fr. 4.3.2 was further purified by RP-HPLC (55% MeOH in H_2_O, 2.0 mL/min) to afford **3** (6.3 mg, tR 14.5 min) and **4** (0.6 mg, tR 21.0 min). 

**Compound** (±)-**1**: Red crystal, m.p. 172–173 °C (MeOH); UV (MeOH) (λ_max_) 224, 325 and 458 nm; ^1^H and ^13^C NMR spectroscopic data, see Table 1; HR-ESIMS m/z 363.0512 ([M − H]^−^, C_20_H_1__1_O_7_, calcd 363.0510). Crystal data: C_20_H_12_O_7_, M_r_ = 364.30, Triclinic, a = 7.4538 (5) Å, b = 12.9674 (7) Å, c = 15.7108 (10) Å, α =89.036 (5)°, β = 89.417 (5)°, γ = 80.470 (5)°, V = 1497.34 (16) Å^3^, space group P-1, Z = 4, D*_x_* = 1.616 mg/m^3^, μ (Cu Kα) = 1.051 mm^−1^, and F (000) = 752. Crystal dimensions: 0.32 mm × 0.24 mm × 0.15 mm. Independent reflections: 5396 (R_int_ = 0.0287). The final R_1_ values were 0.0356, wR_2_ = 0.0875 [I > 2σ(I)]. The X-ray diffraction data were collected at 150 K on an Gemini S Ultra (Oxford Diffraction Ltd., Oxfordshire, UK) with Cu Kα radiation (λ = 1.54178 Å). Structures were identified using direct methods (SHELXS-97) and refined using full-matrix least-squares difference Fourier techniques. H atoms bonded to C were placed on geometrically ideal positions by the “ride on” method, while H atoms bonded to O were located by the difference Fourier method and included in the calculation of structure factors with isotropic temperature factors. The crystallographic data obtained were deposited in the Cambridge Crystallographic Data Centre (Cambridge, UK). Copies of the data may be obtained free of charge through an application addressed to The Director, CCDC, 12 Union Road, Cambridge CB2 1EZ, UK (Fax: +44-(0)1223-336033, or E-mail: deposit@ccdc.cam.ac.uk). CCDC number: 979498.

**Compound** (±)-**2**: Red amorphous powder; m.p. >300 °C; UV (MeOH) (λ_max_) 210, 247, 339 and 441 nm; ^1^H and ^13^C NMR spectroscopic data, see Table 1; HR-ESIMS *m*/*z* 379.0461 ([M − H]^−^, C_20_H_1__1_O_8_, calcd. 379.0459).

**Compound 3**: Yellow power; m.p. >300 °C; ${\left[\alpha \right]}_{D}^{20}$ −267.8 (c 0.28, MeOH); UV (MeOH) (λ_max_) 225, 260 and 342 nm; ^1^H and ^13^C NMR spectroscopic data, see Table 2; HR-ESIMS *m*/*z* 427.0669 ([M − H]^−^, C_21_H_1__5_O_10_, calcd. 427.0671).

**Compound 4**: Yellow power; m.p. >300 °C; ${\left[\alpha \right]}_{D}^{20}$ −681.8 (c 0.11, MeOH); UV (MeOH) (λ_max_) 215, 268 and 354 nm; ^1^H and ^13^C NMR spectroscopic data, see Table 2; HR-ESIMS *m*/*z* 429.0824 ([M − H]^−^, C_21_H_1__7_O_10_, calcd. 429.0827).

### 3.4. Materials and Methods for mPTPB Assay

#### 3.4.1. Cloning, Expression and Purification of MptpB

The full-length PTPB gene was amplified from genomic DNA of the *Mtb* H37Rv strain (School of Life Sciences, Sun Yat-sen University: Guangzhou, China). PCR products were cloned in frame with an *N*-terminal His6 tag into the pET28a (+) vector (Novagen, Merck KGaA, Darmstadt, Germany). For protein expression, plasmids were transformed into *Escherichia coli* BL21(DH3) cells (Invitrogen, Thermo Fisher Scientific, Inc., Hudson, NH, USA) and grown in LB medium containing 50 μg/mL kanamycin at 37 °C till the OD_600_ of the solution was about 0.6. After the addition of 0.1 mM IPTG, the culture was grown for another 16 h at 20 °C. The cells were harvested by centrifugation at 5000 rpm for 5 min at 4 °C. The bacterial cell pellets were resuspended in the buffer containing 20 mM Tris, pH 7.9, 500 mM NaCl, 5 mM imidazole and were lysed by sonication on ice. Cellular debris was removed by centrifugation at 10,000 rpm for 30 min at 4 °C. The protein was purified from the supernatant using glutathione-Sepharose 4B (GE Healthcare, Buckinghamshire, UK), according to the manufacturer’s instructions. Protein concentration was determined using the Bradford dye binding assay (Bio-Rad Laboratories, Inc., Hercules, CA, USA), according to the manufacturer’s recommendations, with bovine serum albumin as the standard. The purified MptpB was stored in 20% glycerol at −20 °C.

#### 3.4.2. MptpB Inhibition Assay

The inhibition assays were performed using the RediPlate 96 EnzChek Tyrosine Phosphatase Assay kit (Invitrogen, Thermo Fisher Scientific, Inc., Hudson, NH, USA) by monitoring the hydrolysis of the fluorogenic phosphatase substrate, 6,8-difluoro-methylumbelliferyl phosphate (DiFMUP), according to the manufacturer’s instruction. The IC_50_ value was determined at five different substrate concentrations by non-linear regression fitting of the inhibitor concentration versus inhibition rate. All measurements were done in triplicate from three independent experiments. The reported IC_50_ were the average value of three independent experiments.

## 4. Conclusions

*Aspergillus* sp. 16-5C, an endophytic fungus from the South China Sea, was proven as a prolific producer of bioactive metabolites. Two unusual pairs of enantiomers of dinaphthalenone derivatives and two new azaphilones, together with four known compounds, were isolated from this fungal strain, and the structures of **1**–**8** were elucidated primarily by NMR experiments. The structure of **1** confirmed by single-crystal X-ray diffraction analysis, while **3** was determined by the optical rotation and **4** was assigned on the basis of the CD and NOESY spectra. The biosynthetic pathways for all these compounds were proposed and suggested that the secondary metabolites are the product of a fungal polyketide synthase. In the bioassay, the isolated compounds were evaluated for their inhibitory activity against MptpB; compounds **1**, **2** and **6** exhibited strong inhibitory activity that suggested they could represent a new type of lead compounds for the development of new anti-tuberculosis drugs.
